# FORMIGA: a fleet management framework for sustainable human–robot collaboration in field robotics

**DOI:** 10.3389/frobt.2025.1706910

**Published:** 2026-01-08

**Authors:** Beril Yalcinkaya, Micael S. Couceiro, Salviano Soares, António Valente

**Affiliations:** 1 School of Sciences and Technology-Engineering Department, University of Trás-os-Montes and Alto Douro (UTAD), Vila Real, Portugal; 2 CORE R&D Department, Ingeniarius, Lda, Alfena, Portugal

**Keywords:** fleet management system, human-robot collaboration, task coding, human-centric interface design, field robotics

## Abstract

Robotic fleet management systems are increasingly vital for sustainable operations in agriculture, forestry, and other field domains where labor shortages, efficiency, and environmental concerns intersect. We present FORMIGA, a fleet management framework that integrates human operators and autonomous robots into a collaborative ecosystem. FORMIGA combines standardised communication through the Robot Operating System with a user-centered interface for monitoring and intervention, while also leveraging large language models to generate executable task code from natural language prompts. The framework was deployed and validated within the FEROX project, a European initiative addressing sustainable berry harvesting in remote environments. In simulation-based trials, FORMIGA demonstrated adaptive task allocation, reduced operator workload, and faster task completion compared to semi-autonomous control, enabling dynamic labor division between humans and robots. By enhancing productivity, supporting worker safety, and promoting resource-efficient operations, FORMIGA contributes to the economic, and environmental dimensions of sustainability, offering a transferable tool for advancing human–robot collaboration in field robotics.

## 1 Introduction

Field domains such as agriculture, forestry, and construction are under increasing pressure to balance productivity demands with sustainability concerns. Persistent labor shortages, environmental constraints, and the need for safer working conditions highlight the urgency of developing innovative robotic solutions (Gonzalez-de Santos et al., 2020). Field robotics, characterized by autonomous systems capable of continuous operation within coordinated fleets, offers significant potential to improve efficiency, reduce costs, and enhance human wellbeing. These machines, equipped with advanced sensing, autonomy, and learning capabilities, are no longer limited to repetitive or isolated tasks–they can actively complement human expertise. Realizing this synergy requires intentional design that supports multiple levels of collaboration, including labor division, mutual assistance, and joint performance in complex and unpredictable environments.

Human–robot teaming thus provides a nuanced perspective on interaction, moving beyond the traditional view of robots as tools for routine work (Gombolay et al., 2015). As robots assume more complex and autonomous roles, it becomes critical to integrate human operators into decision-making processes, ensuring safety, adaptability, and trust (Hoffman and Breazeal, 2004). However, seamless collaboration remains challenging. Autonomy exceptions such as localization errors, navigation failures, or mechanical disruptions can interrupt operations and limit the effectiveness of fully automated systems. To address these limitations, new frameworks are needed that actively support human–machine collaboration rather than relying exclusively on robot autonomy. Progress in this direction depends on close collaboration among researchers, roboticists, and end-users to create systems that are not only technologically advanced but also resilient, transparent, and attuned to human needs in real-world environments (Sikand et al., 2021).

Building on these challenges and aspirations, we introduce the Framework for Optimised Robotic Management, Integration, Guidance, and Automation (FORMIGA^1^). FORMIGA is designed to manage fleets of heterogeneous agents (both robots and humans) by combining optimized operational management, seamless integration, and user-centered guidance. Its architecture incorporates standardized communication through the Robot Operating System, intuitive interfaces for supervision and control, and novel large language model–based task generation. By embedding human oversight into fleet scheduling and task execution, FORMIGA provides a concrete design methodology for effective, sustainable HRC in field robotics.

Accordingly, this research is guided by the hypothesis that a modular, ROS-based fleet management framework integrating human oversight and automated task generation can enhance efficiency, reduce operator workload, and promote sustainable human–robot collaboration in unstructured field conditions.

To examine this hypothesis, the study pursues the following objectives:

1. To design and implement the FORMIGA framework for coordinated management of human and robotic agents in dynamic, unstructured field operations.
2. To evaluate the framework’s performance in simulation through quantitative comparison of autonomous and semi-autonomous operation modes, focusing on execution time and operator workload.
3. To assess the reliability and efficiency of LLM-generated task code before and after fine-tuning, measuring improvements in execution success rate.
4. To demonstrate the framework’s transferability and feasibility under real-world conditions through preliminary field trials within the FEROX project.

## 2 Literature review

The integration of human expertise with robotic capabilities is increasingly essential for achieving sustainable operations in challenging domains. Robots are not only autonomous agents but also components of collaborative ecosystems where human judgment ensures safety, efficiency, and ecological sensitivity (Goldberg, 2019). This balance between autonomy and supervision is particularly relevant in agriculture, forestry, and other field domains, where dynamic and unpredictable environments demand human–robot collaboration to ensure both performance and societal acceptance (He et al., 2021). Effective fleet management systems (FMS) are therefore indispensable, as they orchestrate the planning, allocation, and coordination of humans and robots working together toward shared goals.

### 2.1 Fleet management systems

FMS provide the operational backbone for managing multiple agents, ensuring that robots and humans can act cohesively. Their contribution lies in improving productivity, reducing costs, and enhancing resilience in dynamic settings (Michalec et al., 2021). In field robotics, FMS must support six key features: task formulation, task allocation, coordination, optimization, adaptability, and resilience. Together, these define whether a system can meet real-world sustainability requirements by optimizing resource use, maintaining safety, and ensuring robustness under uncertainty.

- Task Formulation. Traditionally dependent on human expertise (Antunes et al., 2016), task formulation becomes more complex in multi-robot systems where both robot capabilities and human input must be considered (Michalos et al., 2018). Recent advances explore Large Language Models (LLMs) for task specification, producing flowcharts, behavior trees, or executable code (Singh et al., 2023; Ao et al., 2024). While promising, these methods largely remain within structured industrial contexts. More holistic frameworks, such as AUSPEX for UAV decision-making (Döschl et al., 2025), demonstrate how modular, open-source architectures can combine planning, perception, and human-in-the-loop interfaces to enhance flexibility in real-world rescue operations. However, such advances are still rare in terrestrial fleet management, highlighting a gap for field robotics.
- Allocation. Allocation strategies distribute tasks based on agent capabilities, workload, and constraints (Khamis et al., 2015; Matarić et al., 2003). Centralized, decentralized, market-based, and optimization-based paradigms each address aspects of this challenge (Zlot and Stentz, 2005; Zhang et al., 2012; Khamis et al., 2011; Darrah et al., 2005), but their integration into adaptable FMS for heterogeneous, human-inclusive teams remains incomplete.
- Coordination. Coordination ensures synchronized multi-robot operation and prevents conflicts (Yan et al., 2013). Approaches range from potential fields (Vail and Veloso, 2003) to coalition-formation frameworks like CoMutaR (Shiroma and Campos, 2009), and recent taxonomies emphasize static/dynamic and centralized/decentralized distinctions (Verma and Ranga, 2021). Yet, human-centered coordination remains underdeveloped.
- Optimization. FMS often apply multi-objective optimization for energy, time, and resource management (Chakraa et al., 2023; Shelkamy et al., 2020; Karlsson et al., 2007; Tolmidis and Petrou, 2013). However, optimization rarely accounts for the role of humans as active collaborators rather than external supervisors.
- Adaptability. Adaptive planning allows FMS to respond to changing environments (Bolu and Korçak, 2021; Couceiro et al., 2012; Mina et al., 2020). While these advances enhance flexibility, few systems explicitly integrate human adaptability alongside robotic adaptability in dynamic field settings.
- Resilience. Beyond robustness, resilience emphasizes recovery and reconfiguration during disruptions (Couceiro et al., 2014; Prorok et al., 2021). Real-world deployments, such as Valner et al.’s hospital-based fleet automation using RMF (Valner et al., 2022), illustrate both the promise and challenges of resilience in human-centered environments. They demonstrate the value of heterogeneous fleets in dynamic, crowded spaces, but also underline the need for frameworks that explicitly integrate human–robot interaction to maintain continuity.

### 2.2 FMS frameworks

Although no existing framework comprehensively encompasses all the features outlined in the previous section, several noteworthy initiatives, both open-source and commercial, illustrate how fleet management systems are evolving. These frameworks vary in scope, maturity, and applicability, but each contributes insights into task allocation, scheduling, coordination, and resilience that inform future development.

One prominent example is NVIDIA Isaac ROS Mission Dispatch and Client,^2^ which provides a standardised open-source framework for efficient task allocation and tracking between ROS 2 robots. Built on the VDA5050 communication standard, Mission Dispatch leverages lightweight MQTT messaging and containerised services, simplifying integration into existing FMS. Its compatibility with connectors from OTTO Motors and InOrbit demonstrates a strong emphasis on interoperability and scalability within industrial robotics.

ROOSTER,^3^ another ROS-based open-source project, extends beyond allocation into scheduling and autonomous navigation. Its Fleet Manager centralises control with visualised fleet/job statuses, while the Mobile Executor (MEx) Sentinel tracks and allocates tasks dynamically. Together with the Job Manager, these components enable flexible prioritisation and efficient task assignment, offering a robust solution for heterogeneous multi-robot operations.

Open-RMF^4^ represents one of the most versatile open-source frameworks, with ROS 2 libraries supporting interoperability, coordination, and resource optimisation. It integrates robots with shared infrastructure (e.g., lifts, doors, passageways), ensuring conflict-free scheduling. Its ecosystem includes the Traffic Editor for map annotation, Free Fleet for third-party developers, and multiple visualisation interfaces. Simulation plugins for Gazebo and Ignition, along with a multi-factor task planner, further enhance its adaptability for structured environments.

Commercial offerings emphasise scalability and cloud integration. InOrbit^5^ provides a robot operations (RobOps) platform with dashboards tailored to operational roles, real-time fleet monitoring, and incident management. Its Control component addresses autonomy exceptions such as localisation failures or navigation issues, while the time capsule feature enables retrospective analysis of fleet behaviour for optimisation and resilience (InOrbit, 2021). Similarly, Meili Robots^6^ integrates diverse fleets through ERP-compatible interfaces, map editing, and traffic control tools (Robots, 2021; Robots, 2022). By supporting HTTP/REST, WebSockets, and MQTT, Meili emphasises interoperability, though its reliance on structured operational contexts limits deployment in unpredictable field domains.

Formant^7^ is another cloud-based FMS, with strong emphasis on **security** (end-to-end encryption, zero-trust authentication) and workflow automation for teleoperation, localisation, and fleet control (Formant, 2022; Formant, 2024b). Its re-localisation feature allows operators to adjust robot poses interactively, enhancing adaptability during navigation. Early applications in agriculture have been reported (Formant, 2024a), but the platform remains optimised for standardised industrial environments rather than unstructured field robotics.

Overall, these frameworks showcase significant progress but also reveal limitations. Open-RMF excels in coordination and adaptability, ROOSTER in scheduling and dynamic task management, while Formant prioritises secure, centralised control. InOrbit and Meili Robots illustrate scalable, cloud-based fleet management but remain constrained by reliance on predefined workflows. NVIDIA Isaac Mission Dispatch is strong in allocation but narrower in scope. The diversity in performance underscores the absence of a unified FMS capable of integrating humans seamlessly, handling autonomy exceptions in real time, and adapting to unstructured environments. These limitations highlight the need for frameworks such as FORMIGA, which combine interoperability, adaptability, and human-in-the-loop task formulation for sustainability-driven field robotics.

### 2.3 Human–robot collaboration and interaction considerations

Human–robot collaboration (HRC) extends beyond mechanical cooperation to encompass cognitive and social interaction, aiming to combine human adaptability with robotic precision for shared task achievement (Bauer et al., 2008). In dynamic domains such as agriculture and forestry, robotic systems often rely on human supervision due to incomplete autonomy, leveraging human cognitive abilities for decision-making and adaptation (Adamides et al., 2017). User interface (UI) design therefore plays a central role in shaping how operators perceive, command, and trust their robotic partners. Studies have shown that multimodal interfaces such as gesture, voice, or touch-based control facilitate intuitive communication, while adaptive UIs can lower operator workload and enhance safety by maintaining situational awareness (Adamides et al., 2017; Drury et al., 2003).

Usability is a foundational aspect of effective human–robot collaboration, determining how easily and efficiently operators can interact with robotic systems to accomplish their tasks. Schraick et al. (Schraick et al., 2025) evaluated collaborative robots in forestry and agroforestry scenarios and demonstrated that high usability, measured using the System Usability Scale (SUS), not only improves productivity and safety but also reinforces the importance of human-centered and accessible design in AI-driven robotic systems. Trust and transparency are equally crucial, as operators must understand robot intentions and system states to calibrate oversight effectively (Wright et al., 2019). Recent work on proactive HRC further highlights the need for bi-directional cognition, where humans and robots dynamically exchange roles and intentions to achieve fluent teamwork under uncertainty (Li et al., 2021). Collectively, these insights emphasize that sustainable field robotics requires not only robust automation but also human-centered design, embedding transparency, usability, situational awareness, and trust within the operational interfaces and collaboration models adopted by emerging frameworks.

## 3 Methodology: FORMIGA framework

FORMIGA is a fleet management system designed to coordinate both robotic and human agents in dynamic field environments. It provides a unified platform for multi-agent supervision, task authoring and execution, and resource-aware allocation with human-in-the-loop oversight.

FORMIGA was developed through a structured design process that builds upon the ROOSTER framework. ROOSTER, an open-source ROS project, contributes core FMS capabilities (task allocation, scheduling, and navigation).

**FIGURE 1:**
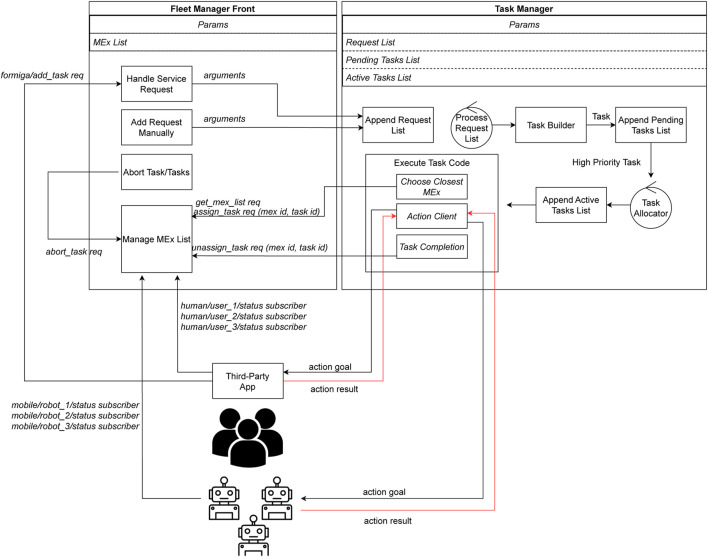
FORMIGA architecture. Major components: ROS messaging backbone; Fleet Manager Front (UI/API); Task Manager (Task Builder, Allocator, Queues); Action Clients; third-party apps for human agents.

Figure 1 shows an overview of the FORMIGA ecosystem, highlighting key components that optimize fleet management. FORMIGA extends ROOSTER’s base with features required for unstructured field scenarios and human–robot teaming: (i) a *Mobile Executor* (MEx) model that treats humans and robots uniformly; (ii) standardized topics, services, and actions across agent types; (iii) a task-coding pipeline that composes ROS actions; and (iv) a large language model (LLM) module that generates task code from natural language.

### 3.1 ROS integration and standardisation

A central design principle of FORMIGA is the standardisation of communication between heterogeneous agents–robots and humans–through the Robot Operating System (ROS). ROS provides a flexible framework for modular robot software, and in FORMIGA it is used to ensure interoperability across diverse agents and applications.

#### 3.1.1 Namespaces

FORMIGA adopts a consistent naming convention to avoid conflicts and clarify responsibilities within the multi-agent system. Namespaces follow the pattern agent_type/agent_name/status, with examples such as:

- /ground_robot/robot_1/status
- /aerial_robot/robot_2/status
- /human/user_1/status

This convention ensures transparent identification of both robotic and human executors, enabling uniform handling of their data streams.

#### 3.1.2 Topics: MEx status

ROS topics support publish–subscribe communication among distributed nodes. FORMIGA subscribes to the *status topic* of each MEx–whether a robot or a human bridged via a third-party app–capturing availability, pose, battery levels, and other vital information. FORMIGA augments this data with fleet-level details, such as current task assignment, enabling real-time task allocation and mutual assistance scenarios where human judgment complements robot autonomy.

**FIGURE 2:**
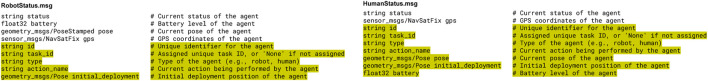
Structure of MexStatus.msg for Mobile Executors (MExs) in FORMIGA. Highlighted fields are added at the fleet-management level.

Figure 2 illustrates the structure of MexStatus.msg, a custom ROS message defined for FORMIGA but reusing community-standard types (e.g., geometry_msgs/Pose, sensor_msgs/NavSatFix). This ensures compatibility while providing the specific data structures needed for human–robot integration.

#### 3.1.3 Services: task request

ROS services provide synchronous, request–response interactions. FORMIGA exposes services to enable both internal modules and external applications to add, cancel, assign, or unassign tasks. Examples include:

- /formiga/add_task: request a new task with parameters such as priority (low, medium, high), agent name, and arguments;
- /task_manager/abort_task: cancel an ongoing or pending task;
- /fleet_manager_front/get_mex_list: retrieve the status of all active agents;
- /fleet_manager_front/assign_task: allocate a task to a specific MEx;
- /fleet_manager_front/unassign_task: release an agent and revert its status to standby.

These services ensure direct interaction and immediate feedback, while allowing human knowledge–via the user interface or third-party apps–to shape robot autonomy within ROS-standard conventions.

#### 3.1.4 Actions: task decomposition

ROS actions are designed for long-running behaviours that require feedback and possible preemption. In FORMIGA, tasks are decomposed into sequences of ROS actions, with action clients in FORMIGA sending goals to agent-side action servers. Features include:

- Goal management: tasks are issued as goals to robots or humans, with continuous feedback on progress;
- Preemption: tasks can be interrupted if higher-priority operations arise or if human operators intervene;
- Feedback and result handling: FORMIGA monitors intermediate updates and final outcomes to decide on continuation, reassignment, or termination.

During execution, an agent’s status transitions through states (ASSIGNED, EXECUTING ACTION, etc.) and is updated in real time. Successful completion returns the agent to standby, whereas failures trigger task abortion.

### 3.2 User interface

**FIGURE 3:**
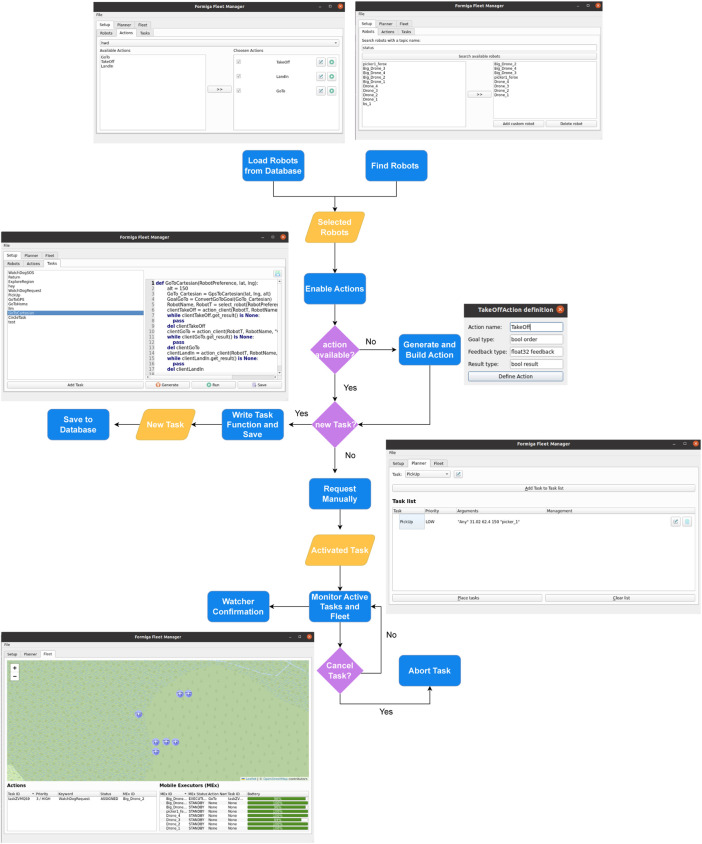
User flow of the FORMIGA interface, illustrating the streamlined process for managing and integrating human-robot teams efficiently.

The FORMIGA interface is designed to be intuitive and operator-centred, ensuring efficient configuration, supervision, and control of heterogeneous human–robot teams. It not only functions as an operational dashboard but also serves as a mediator of transparent and collaborative interactions between humans and autonomous agents. Figure 3 illustrates the overall user flow, from system setup through task execution.

#### 3.2.1 Fleet setup and monitoring

Integrating new agents into FORMIGA is streamlined to minimise operator workload. By querying the network with the keyword status, all active agents are automatically listed. Both robotic agents and human operators (connected via third-party mobile apps) are detected and classified into types such as ground robot, aerial robot, or human, using the namespace convention described in Section 3.1. Fleets previously stored in a SQLite database can also be reloaded for rapid deployment.

Once added, FORMIGA scans each agent for available action servers and integrates them into the fleet. This process ensures that even previously unknown agents can be incorporated on demand.

During operation, the interface provides real-time monitoring of:

- MEx statuses such as availability, pose, battery levels, and current task;
- Service interactions, including task requests, assignments, and cancellations;
- Action execution updates with detailed progress and outcomes.

This data is presented through a Leaflet-based visualisation that overlays OpenStreetMap (OSM) maps with a live task pipeline. Operators can track unique task IDs, priority levels (LOW, MEDIUM, HIGH), task names, current status (PENDING, ACTIVE, ASSIGNED, ABORTED, SUCCEEDED), and agent assignments. Such transparency supports both supervision and occasional operator intervention, strengthening human-in-the-loop control in field deployments.

#### 3.2.2 Task coding: manual and automated

Tasks in FORMIGA are defined as Python functions composed of ROS action clients, each sending goals to action servers hosted by robots or human third-party app. This approach allows sequential or parallel execution of behaviours and ensures flexibility in mission design. Task functions run in background threads, keeping the interface responsive and enabling multiple tasks to be executed concurrently.

**FIGURE 4:**
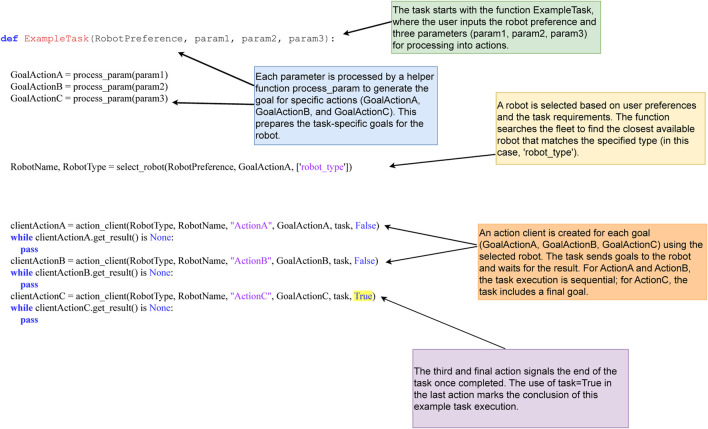
Example of a user-defined Python task in FORMIGA, demonstrating agent selection and sequential action execution.

A typical task function begins by setting parameters, selecting an agent (either explicitly chosen by the user or automatically via FORMIGA’s select_robot function), and issuing a series of actions. Results are monitored in real time; failures trigger resource release and optional reassignment. Figure 4 illustrates an abstract example with three sequential actions.

The action_client function, which sends action goals to selected agents, follows a standardised format:

action_topic = “/” + RobotType + “/” + RobotName + “/action/” + “/ActionA/”

While manual coding allows fine-grained control, the effort grows quickly with task complexity. To address this limitation, FORMIGA integrates a Large Language Model (LLM) module (see Section 3.4) that converts natural language prompts into executable Python task functions. This reduces operator workload, accelerates mission specification, and enhances system adaptability in unstructured environments where rapid re-tasking is required.

### 3.3 Task management

FORMIGA builds on ROOSTER’s robust task management system, enabling efficient definition, allocation, and execution of tasks to facilitate multi-agent collaboration and dynamic resource optimisation. The process begins when the Task Manager node receives a task request and appends it to the global order list. A callback function, Task Builder, processes this list every second, constructing tasks from the provided parameters and adding them to the Pending Tasks List.

Once tasks are ready, the Task Allocator retrieves the highest-priority task and moves it to the Active Tasks List for execution. During task execution,the user-defined or LLM-generated task code is initiated, which includes a call to the select_robot function. This function assesses user preferences and the type of actions required, subsequently requesting an updated MEx list from the Fleet Manager. It then invokes the choose_closest_mex function, which filters available MEx units with a status of STANDBY, matching the task’s requirements. This function calculates the distance to the target location for each available agent and selects the closest MEx based on this distance, ensuring that the optimal agent is chosen to minimise response time and enhance operational efficiency.

After selecting the robot, an assign_task service request is sent to the Fleet Manager Front module. The selected robot’s status transitions from STANDBY to ASSIGNED, marking its readiness for the task.

Once all actions within the task are executed, the system carefully manages task completion and status updates. If the task is successfully completed, the robot’s status returns to STANDBY, and the task is removed from the Active Tasks List. In the case of failure or abortion, the task status is updated to ABORTED, triggering the task completion processes to ensure reliable management of ongoing operations.

### 3.4 LLM-generated task coding

In recent years, the rise of Large Language Models (LLMs) has demonstrated remarkable potential for generating executable code, enabling advancements in general planning. This aligns perfectly with the dynamic nature of human-robot applications, where the generation of new, context-specific tasks is often necessary. LLMs, trained on vast programming corpora, have been shown to perform well in generating pythonic structures, as evidenced by state-of-the-art studies, like ProgPrompt (Singh et al., 2023). In the FORMIGA ecosystem, we leverage Large Language Models (LLMs) to automate and streamline the development of complex robotic behaviours, reducing the need for extensive manual coding. As presented in the previous section, FORMIGA leverages the structured nature of Python to model tasks as a sequence of actions. This concept is directly compatible with LLMs’ ability to understand and generate pythonic functions. This integration not only streamlines the task-coding process, but also augments FORMIGA’s adaptability, allowing it to handle unforeseen operational challenges without extensive manual intervention. Consequently, this integration supports the execution of collaborative actions in real-world scenarios, enhancing the effectiveness of human-robot interaction within the ecosystem.

**FIGURE 5:**
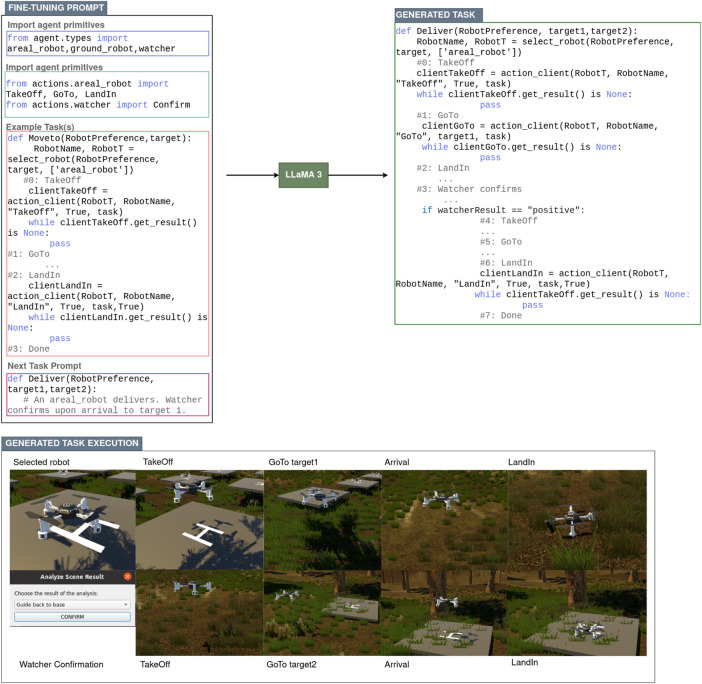
LLM-generated task execution with LLama3.

Figure 5 depicts a general overview on how LLM-generated task coding is integrated within FORMIGA. In the following subsections, we delve into the specifics of the LLM framework adopted in FORMIGA, the methodology behind constructing programming language prompts for task generation, and insights into the actual task coding process.

#### 3.4.1 LLM framework

Integrating an LLM directly within FORMIGA provides the opportunity to harness powerful language models for task coding without relying on external infrastructure. However, a local deployment of an LLM, particularly models like GPT-4 or LLaMA 3, demands high computational power, often necessitating specialised hardware, such as NVIDIA A100 GPUs or custom AI accelerators. The hardware and energy costs associated with maintaining such infrastructure can be prohibitive, especially when targeting on-field human-robot operations, where mobile and compact computing solutions are preferred. Given these constraints, we opted to explore existing cloud-based LLM frameworks that offer high-performance computing without the need for costly local hardware setups.

Some potential alternatives to local LLM integration include:

- OpenAI API: Provides access to powerful models like GPT-4 via a cloud-based subscription service, making it possible to generate and fine-tune code with minimal local computational requirements.
- Hugging Face Inference API: Offers various state-of-the-art models, including the LLaMA series, in a flexible environment that allows developers to use models on-demand, with a focus on open-source solutions.
- Azure OpenAI Service: Integrates Microsoft Azure’s infrastructure with OpenAI’s language models, enabling scalable and secure access to LLMs within enterprise environments. This platform also includes specific optimisations for high-performance computation and cloud-based deployment.
- Groq Platform: A specialised cloud platform designed to work seamlessly with large-scale language models, providing low-latency access to AI capabilities and supporting the robust computational needs required for real-time task generation in robotics.

After evaluating these options, Groq^8^ was chosen as the computing platform for FORMIGA due to its focus on handling high-throughput, low-latency AI computations that align with the operational demands of the fleet management system. In conjunction with the LLaMA 3 model, specifically the llama3-70b-8192 configuration, Groq’s infrastructure excels in generating sophisticated Python code as required by FORMIGA. The llama3-70b-8192 model, with its 70 billion parameters, offers an exceptional balance between complexity and efficiency, ensuring that FORMIGA can generate reliable and context-aware task codes dynamically. Groq’s platform not only meets the high computational requirements for real-time code generation but also offers the flexibility needed for integrating LLM-generated code directly into the ROS-enabled ecosystem, making it the optimal choice for our framework.

#### 3.4.2 Programming language prompts

To optimise the LLaMA 3 model for our specific use case, we engaged in a process of fine-tuning approach by drawing inspiration from methodologies, such as ProgPrompt (Singh et al., 2023). This fine-tuning was aimed at adapting the model to the domain-specific requirements of the FORMIGA ecosystem, advocating for a more structured approach to fine-tuning, where the model is trained not only on a wide variety of tasks, but also on programmatic specifications of available agents within an environment and related actions, enabling it to generate Python code that is both functionally correct and contextually appropriate for human-robot collaborative tasks.

The fine-tuning process was designed to help the model internalize the specific coding conventions and logical structures used within FORMIGA. We began by prompting the model with information on available agent types and their capabilities in the FORMIGA ecosystem, ensuring it understands which types of robots or humans can execute specific actions. Next, we provided action primitives–fundamental operations such as move, patrol, or load package–presented as Python functions to train the LLM in recognizing valid commands within the system. By integrating examples of the FORMIGA API, such as how actions are translated into commands for agents via ROS, we ensured seamless compatibility with FORMIGA’s architecture. These examples also illustrated how tasks are constructed from basic actions, highlighting the relationship between function definitions and action sequences. For example, a task might instruct a drone to take off, patrol an area, and land, with the LLM generating a corresponding function that, when executed, sends these commands to the relevant agents.

By training on these examples, the model learned how to initialise parameters, interact with ROS action servers, and handle errors in a way that aligns with the operational needs of the FORMIGA ecosystem.

#### 3.4.3 Task coding generation

Once fine-tuned, the model can be queried to generate new task functions that were not part of the initial training set. This capability is particularly valuable when a novel or unanticipated task needs to be coded quickly. For instance, the model can interpret a high-level goal provided by the user, map it to the appropriate agent actions, and generate Python code that defines the new task. The model automatically identifies necessary parameters, selects the suitable agent based on the context or user input, and establishes interaction with the ROS action server for execution. Figure 5 exemplifies the kind of task structure the model can generate, highlighting how it translates user intent into executable code compatible with the FORMIGA framework. In such cases, the LLM ensures that the generated code integrates seamlessly into the system, allowing concurrent execution with other tasks.

During generation, the model occasionally produced invalid commands or referenced functions not defined within the FORMIGA framework. When such code is executed, Python raises exceptions, preventing the task from starting. This mechanism effectively exposes errors before execution, ensuring that faulty code can be identified and corrected.

## 4 Case study: the FEROX project

FEROX^9^ is a project that aims to support workers collecting wild berries and mushrooms in the remote and challenging terrains of Nordic countries through the use of robotic technologies. The project places a strong emphasis on Human–Robot Collaboration by deploying unmanned aerial vehicles (UAVs) to monitor and assist groups of workers during their field operations. This approach enhances worker safety in remote environments where access to help or assistance may be limited.

The anticipated outcomes include increased worker trust in collaborating with robots, higher yields of harvested berries, improved product quality for consumers, more efficient picking times, enhanced worker safety in remote locations, and reduced levels of worker exhaustion.

**FIGURE 6:**
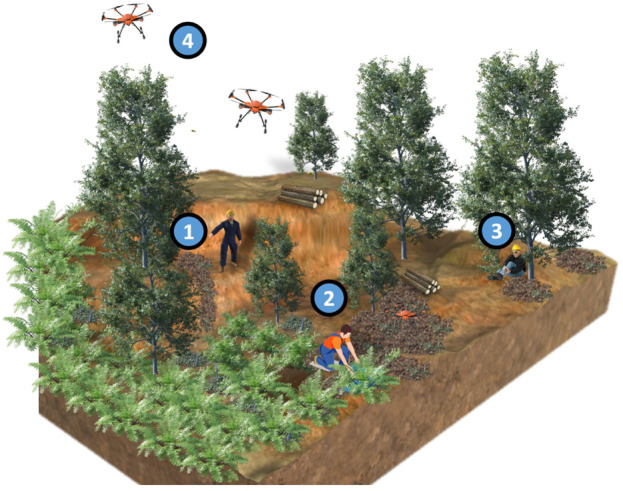
An overview of the FEROX use case scenario. 1) The fleet manager coordinates humans and drones through FORMIGA and confirms triggers raised by robots; 2) Human pickers collect berries using the PickerApp for guidance and to request drone services; 3) Pickers can request tasks (e.g., WatchDogRequest) or have tasks automatically triggered (WatchDogSOS); 4) UAVs include Light Weight Drones (LWDs) for reconnaissance and High Weight Drones (HWDs) for assisting humans during picking.

Figure 6 illustrates the conceptual architecture of FEROX. The project also addresses dimensions beyond the scope of this paper, including AI methods for berry yield identification and mapping (FBK, 2024), AI methods for human activity recognition (Yalcinkaya et al., 2024b), and human factors, standards, and ethics (Fletcher et al., 2023). Within this broader ecosystem, FORMIGA serves as the fleet management backbone, orchestrating both robot and human actions to enable adaptive, context-aware task execution.

### 4.1 FEROX simulator

**FIGURE 7:**
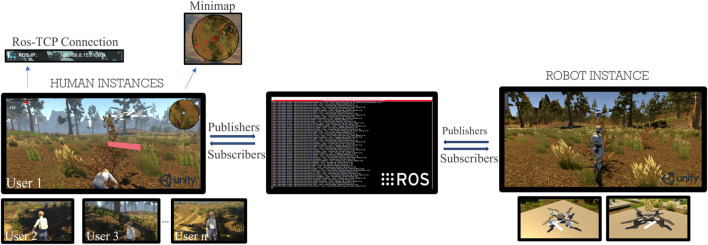
General overview of the FEROX simulator architecture.

The FEROX simulator provides a controlled virtual environment for replicating berry-picking scenarios, combining Unity’s physics and immersive rendering with ROS’s robotics integration (Yalcinkaya et al., 2024a). The ROS-TCP Connector^10^ ensures bidirectional communication, synchronising operator actions with robotic behaviours across distributed instances (Figure 7).

Two instance types enable both single- and multi-agent HRC scenarios:

- Robot Instance (Backend): Runs ROS on Ubuntu, managing UAVs represented as Unity NavMesh Agents. Robots autonomously navigate, interact with dynamic elements (e.g., berry spawning), and execute ROS services. Two UAV types are supported: Light Weight Drones (LWD) for reconnaissance and mapping, and High Weight Drones (HWD) for logistics, navigation support, and emergencies.
- Human Instance (Frontend): Each player controls a 3D picker avatar in Unity, connected to ROS in real time. Pickers harvest berries, request UAV support, and load payloads, following a multiplayer-game paradigm.

Both LWDs and HWDs provide ROS action servers (TakeOff, GoTo, LandIn); LWDs additionally support Explore. Clients (human or automated) trigger these actions, with real-time feedback ensuring UAVs adapt to environmental conditions.

### 4.2 Human-mediated operations

Human-mediated operations are central to the FEROX workflow. FORMIGA supports collaborative execution by integrating operator requests with autonomous UAV capabilities.

**FIGURE 8:**
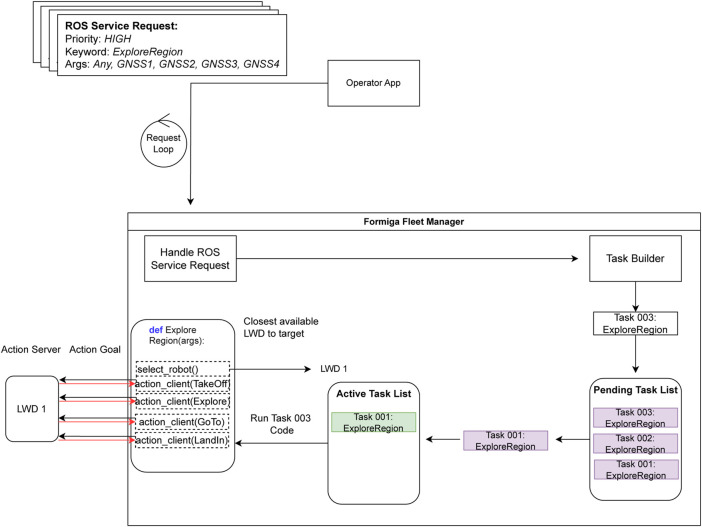
Workflow of FORMIGA’s ExploreRegion task for reconnaissance.

The reconnaissance phase precedes berry picking. LWDs execute the ExploreRegion task, autonomously scanning large areas to generate semantic maps for or forestry inventory. The Operator App segments fields into polygons, each represented as GNSS coordinates, which are submitted to FORMIGA as ROS service requests. FORMIGA schedules these tasks, allocates the nearest available LWD, and monitors execution (Figure 8). This workflow ensures efficient coverage while accounting for UAV constraints such as battery life.

**FIGURE 9:**
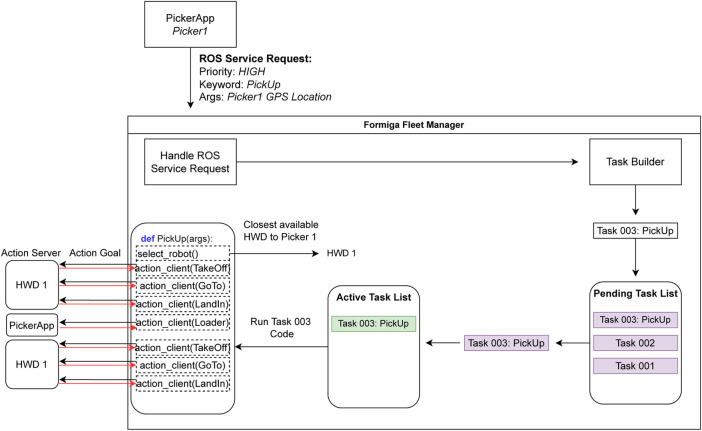
Workflow of FORMIGA’s PickUp task during berry collection.

Following reconnaissance, the Picking phase combines logistics and safety tasks (PickUp, WatchDogSOS, WatchDogRequest). UAVs collaborate directly with pickers (via the PickerApp) to transport harvested berries, respond to emergencies, and guide lost human workers (Figure 9). The integration of these tasks demonstrates FORMIGA’s ability to blend human agency and UAV autonomy in real time, ensuring both efficiency and worker safety.

## 5 Experimental evaluation

In this section, we present the experimental setup and results used to assess two complementary aspects of the FORMIGA framework: (i) baseline performance of the fleet management system in supporting human-mediated autonomy, and (ii) the effectiveness of LLM-generated task coding for novel task scenarios in the FEROX simulator.

### 5.1 FORMIGA baseline performance

The first experiment evaluated FORMIGA’s ability to optimise execution flow and reduce operator workload by comparing two modes of operation:

1. Autonomous: FORMIGA selects drones, schedules actions, and manages task execution automatically.
2. Semi-autonomous: The human operator manually selects drones, enters parameters, and triggers each action in sequence.

The PickUp task was chosen as the benchmark. In this task, a HWD flies to a picker, waits while berries are loaded, and returns to base. A total of 10 trials were conducted in each mode. The execution flow includes the following actions:

1. Drone selection (closest HWD).
2. TakeOff.
3. GoTo: Navigate to picker location.
4. LandIn.
5. Wait for human confirmation of loading.
6. TakeOff.
7. GoTo: Return to base.
8. LandIn.

In the semi-autonomous mode, the operator was responsible for manual drone selection, entering target coordinates, and sequentially triggering each action. This process introduced idle time and additional cognitive load compared to the fully autonomous execution.

**FIGURE 10:**
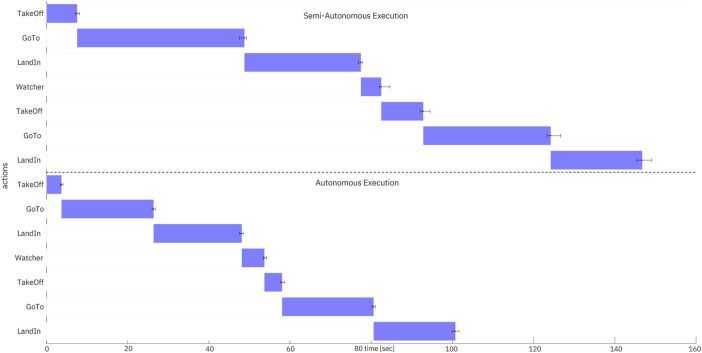
Comparison of autonomous vs. semi-autonomous execution times for the PickUp task.

A box-plot–based visualisation (Figure 10) was employed to represent both the temporal sequence of task actions and the variability in their execution times across trials with the quartile distribution of a standard box plot.

Results show that the autonomous system reduced total execution time by 37.2% compared to semi-autonomous operation. FORMIGA’s automated sequencing eliminated idle delays between actions, resulting in smoother task flow. Moreover, variability in execution time was significantly lower in autonomous trials, underscoring improved consistency and predictability.

These findings highlight FORMIGA’s effectiveness in minimising operator effort while ensuring reliable task management–critical for scaling operations in complex, human-robot collaborative environments such as berry picking. By reducing idle time and execution variability, the system also promotes more sustainable operations, both by lowering energy consumption of robotic agents and by alleviating the cognitive load on human operators.

### 5.2 Evaluation of LLM-generated tasks

The second experiment assessed the ability of a fine-tuned LLM to generate executable code for novel tasks within the FEROX simulator. Following the methodology in Section 3.4, the evaluation involved two phases:

1. Fine-tuning on a set of five predefined tasks, $T_{\mathrm{pre}}=\left\{T_{1},\ldots ,T_{5}\right\}$ .
2. Generating and testing five novel tasks, $T_{\mathrm{LLM}}=\left\{T_{6},\ldots ,T_{10}\right\}$ , not included in training.

Each novel task was generated 30 times, yielding 150 candidate codes. The tasks ranged from simple path-following to multi-agent coordination:

- Circle $\left(T_{6}\right)$ : UAV flies in a circle around the base.
- Triangle $\left(T_{7}\right)$ : Three UAVs form a triangle and land together.
- ZigZag $\left(T_{8}\right)$ : UAV reaches target via zigzag trajectory.
- Patrol and Escort $\left(T_{9}\right)$ : One UAV escorts, another patrols in a circle asynchronously.
- Delivery $\left(T_{10}\right)$ : Two UAVs perform asynchronous delivery and return operations.

We evaluated the quality of LLM-generated code using three complementary metrics. Success rate measured the proportion of tasks that executed correctly in the simulator, serving as a direct indicator of functional reliability. CPU time, defined as the interval between request and code generation, reflected computational efficiency and responsiveness. Finally, we employed CodeBLEU (Ren et al., 2020), an adaptation of BLEU for programming languages that accounts for both syntactic and semantic quality. CodeBLEU integrates weighted n-gram matching, which emphasises programming-specific keywords, abstract syntax tree (AST) matching for structural similarity, and data-flow matching to capture semantic correctness through variable dependencies.

### 5.3 Results

**FIGURE 11:**
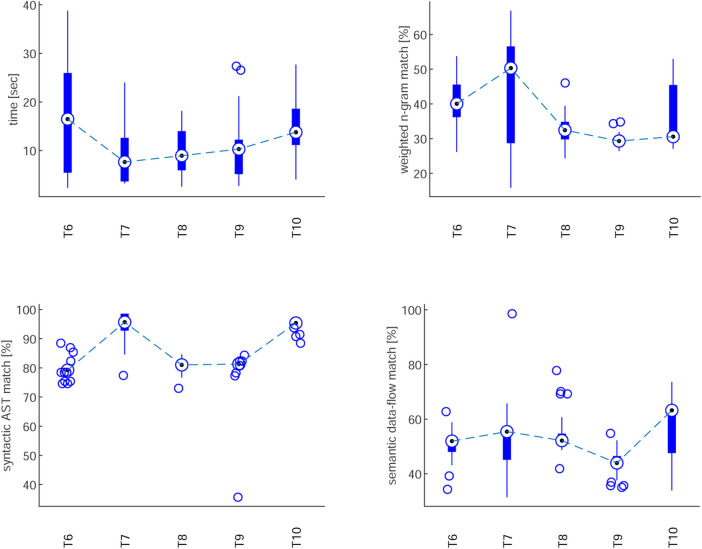
Evaluation of LLM-generated tasks: CPU time and CodeBLEU metrics.

Five unseen tasks ( T6 – T10 ) were generated 30 times each, yielding 150 variants. Table 1 shows success rates, while Figure 11 illustrates CPU time and CodeBLEU scores across 30 generations per task, summarised using descriptive statistics. Each box plot displays the median as the central mark, with the bottom and top edges representing the 25th and 75th percentiles, respectively, to visualise variability in performance across trials.

**TABLE 1:** Accuracy of LLM-generated tasks.

Task	$T_{6}$	$T_{7}$	$T_{8}$	$T_{9}$	$T_{10}$
Accuracy	93.3%	76.7%	73.3%	53.3%	53.3%

The evaluation revealed notable differences across tasks of varying complexity. Accuracy ranged from high success in simple tasks (e.g., T6 : 93.3%) to substantially lower rates in more complex scenarios (e.g., T10 : 53.3%), underscoring the influence of task complexity on model performance. CPU time followed a similar trend, with straightforward tasks such as T7 requiring minimal processing (7.6 s), while more complex tasks ( T6 , T10 ) demanded longer generation times (13–16 s). Notably, extended computation did not necessarily translate into higher accuracy. Finally, CodeBLEU metrics indicated that simpler tasks achieved higher scores in weighted n-gram and AST matching, whereas more complex tasks, despite maintaining a degree of semantic consistency, frequently failed to produce functionally executable code.

Simpler tasks (e.g., $T_{6}$ , $T_{7}$ ) achieved higher accuracy and shorter CPU times. More complex tasks ( $T_{9}$ , $T_{10}$ ) showed both lower accuracy and reduced CodeBLEU scores, highlighting the challenges of multi-agent coordination and asynchronous execution.

**FIGURE 12:**
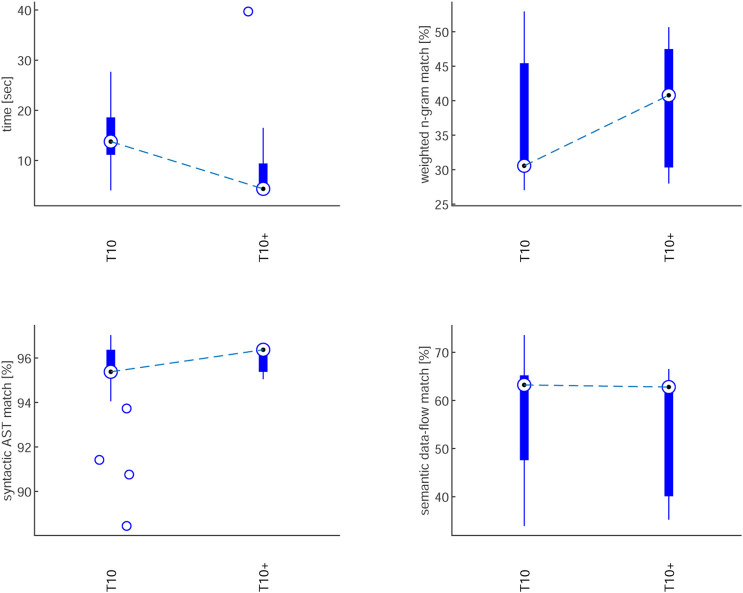
Comparison of $T_{10}$ vs. fine-tuned $T_{10+}$ results.

To improve performance, we fine-tuned the LLM by adding ground-truth code from T6 – T9 and regenerating T10 (T10+) . Accuracy rose from 53.3% to 73.3%, CPU time dropped from 13.8s to 4.3s, and n-gram scores improved (Figure 12). These results confirm that incorporating domain-specific examples enhances both efficiency and reliability for complex task generation.

## 6 Initial field trials and usability

**FIGURE 13:**
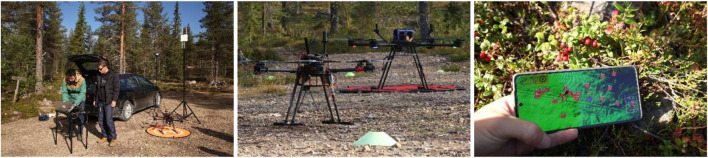
Preliminary field trials in Rovaniemi (Finland). Left) Team managing drones in the forest; Centre) HWDs performing PickUp, WatchdogRequest, and WatchdogSOS; Right) PickerApp interface for requesting services and providing feedback.

The field evaluations compared FORMIGA’s orchestration performance between the 2024 Rovaniemi trials and the 2025 Nuuksio (Mustakorpi) campaigns (Figure 13). Preliminary field trials were conducted in Rovaniemi, Finland (September 2024) to transfer simulator-derived behaviours to real forest conditions. Drones successfully executed the PickUp (HWD-assisted transport), WatchdogRequest, and WatchdogSOS (LWD “guide-home”) tasks with only minimal adaptations. The main challenge encountered was communication reliability: cellular coverage gaps required fallback to peer-to-peer radio links and GNSS recalibration. When the end-to-end workflow, from PickerApp to FORMIGA, was successfully established, missions were completed as planned. In several cases, however, operators had to manually initiate missions when requests could not traverse the network. While such cases were not counted as app-origin successes, they provided valuable insight into field robustness and operator intervention needs.

Follow-up campaigns in Nuuksio (Mustakorpi, July 2025) scaled up operations and human–robot orchestration. Each team included one LWD for Watchdog tasks, one HWD for PickUp, and five experienced berry pickers using the PickerApp, coordinated through FORMIGA. Trials followed a looped route combining mapping, harvesting, drone-assisted transport, and return-to-base operations. Compared to 2024, FORMIGA was enhanced with deterministic acknowledgment handshakes between PickerApp and the fleet manager, retry/queue semantics with duplicate suppression, and refined timeout policies.

Three metrics were assessed: agent concurrency, pick-up workflow success, and watchdog (escort) workflow success. These metrics capture FORMIGA’s ability to coordinate multiple human and robotic agents, maintain reliable task execution, and ensure end-to-end workflow integrity under real-world communication constraints.

Concurrent orchestration was defined as the number of agents, both robots and human operators, managed simultaneously by the fleet management system. Each live session with an assigned task counted as one active agent. The peak concurrency (PCA) was calculated as shown in Equation 1:

$$PCA=\underset{t\in T}{\max}\left(N_{\text{LWD}}\left(t\right)+N_{\text{HWD}}\left(t\right)+N_{\text{PickerApp}}\left(t\right)\right).$$ (1)

During the 2024 baseline trials, FORMIGA coordinated one HWD, one LWD, and one human picker using the PickerApp. In 2025, this scaled to eight concurrent agents (one LWD, one HWD, and six pickers), representing a 2.67 × increase (167%) in operational capacity. This transition reflects FORMIGA’s progression from single-actor demonstrations to coordinated, mixed human–robot team operations.

Pick-up workflows measured FORMIGA’s ability to complete transport requests initiated through the PickerApp, confirmed by the fleet manager, executed by the HWD, and logged upon return. A workflow was considered successful when all intermediate steps–including acknowledgement (ACK), dispatch, arrival, payload handover, and closure–were completed within configured timeouts. The success rate was computed as in Equation 2:

$${Success}^{\text{pickup}}\left(\%\right)=100\times \frac{N_{\text{succ}}^{\text{pickup}}}{N_{\text{valid}}^{\text{pickup}}}.$$ (2)

In 2024, two of three valid requests succeeded (66.7%), whereas in 2025, five of six were successful (83.3%). The observed improvement resulted from the introduction of deterministic ACK handshakes, queue-based retries, and refined timeout policies. Remaining failures were attributed to extended cellular outages rather than software or protocol defects.

Similarly, Watchdog (escort) workflows assessed FORMIGA’s ability to dispatch an LWD in response to SOS or guidance requests from the PickerApp. Success required full completion of the communication and escort pipeline: message acknowledgment, dispatch, arrival, escort initiation, and return confirmation. The success rate was defined as in Equation 3:

$${Success}^{\text{wd}}\left(\%\right)=100\times \frac{N_{\text{succ}}^{\text{wd}}}{N_{\text{valid}}^{\text{SOS}}}.$$ (3)

Results closely mirrored those of the pick-up tasks, improving from 66.7% in 2024 to 83.3% in 2025. As with the previous metric, residual failures occurred under conditions of sustained network loss.

From a usability perspective, the trials also highlighted the importance of intuitive interfaces and clear feedback mechanisms between the PickerApp, operators, and drones. Picker feedback indicated reduced need for manual intervention and faster confirmation of service status, reflecting improved situational awareness and trust in automated scheduling. These findings align with broader evidence that usability and transparency are central to sustained human–robot collaboration, particularly in field contexts where operators must balance supervision with physical tasks (Schraick et al., 2025).

## 7 Discussion and limitations

Experiments in the FEROX simulator confirmed that FORMIGA streamlines execution and reduces operator load. In tasks such as PickUp, autonomous execution reduced duration by 37% compared to semi-autonomous control, while also lowering variability in task completion. This balance of efficiency and consistency is crucial for repetitive, labour-intensive workflows. Furthermore, FORMIGA proved capable of handling asynchronous flows, coordinating drones and humans concurrently, an essential property for scalable operations.

From the end-user perspective, these results directly translate into a more balanced and transparent division of labor between humans and robots. FORMIGA automatically allocates tasks to UAVs, while reserving decision-oriented and supervisory actions for human operators. This reduces the need for manual command input and allows operators to focus on higher-level coordination, safety monitoring, and task prioritization. In the PickUp scenario, for example, the system autonomously selects the nearest available drone and manages the transport cycle, while the human worker only confirms payload readiness through the interface, minimizing idle time and cognitive burden.

Building on these results, the evaluation of LLM-generated code demonstrated that generative AI can support sustainable autonomy by lowering the barrier to programming complex behaviours. While simple tasks were generated reliably, multi-agent scenarios remained more error-prone. However, targeted fine-tuning with ground-truth data improved accuracy by 20% and cut CPU time threefold, highlighting the role of domain adaptation in making LLM integration both effective and resource-efficient.

Despite these improvements, it is important to recognize that CodeBLEU, while a valuable metric for assessing syntactic and semantic similarity, does not fully capture functional correctness or runtime robustness. To address this, all generated code was executed in simulation to validate functional performance, with success rate and CPU time serving as complementary quantitative indicators. Nonetheless, CodeBLEU alone cannot account for operational reliability under real-world conditions, such as fluctuating connectivity or sensor noise.

The integration of the Groq accelerator and fine-tuned LLaMA 3 models enabled efficient execution of language model components while maintaining practical deployment feasibility in the field. However, dependence on cloud infrastructure may introduce latency, connectivity limitations, and potential security risks, especially in remote agricultural or forestry environments. To mitigate these challenges, future work can explore a hybrid local-cloud architecture, where time-critical decision modules and scheduling processes operate locally on edge devices, while more resource-intensive computations are handled in the cloud.

Overall, although limited in scope, these experiments and field trials demonstrated FORMIGA’s readiness for sim-to-real deployment and provided valuable insights for future refinements in networking, scalability, and usability evaluation. The findings also highlight the importance of hybrid autonomy, balancing automated task management with human supervision, to ensure sustainable integration of AI-driven systems in real-world field robotics.

## 8 Conclusion

FORMIGA provides a modular and sustainable framework for human–robot collaboration in unstructured environments. By combining standardized ROS communication with LLM-assisted task coding, it supports both reliable orchestration of routine workflows and flexible adaptation to emerging mission demands.

Within the FEROX project, simulations showed significant efficiency gains and reduced execution variability, while evaluations of LLM-generated task code demonstrated the value of targeted fine-tuning for complex, multi-agent missions. Initial field trials validated FORMIGA’s ability to operate under real-world constraints, confirming its potential to enhance safety, reduce physical strain, and enable more sustainable workforce practices in domains such as agriculture and forestry.

A key strength of FORMIGA lies in its open-source commitment and modular ROS-based architecture, which ensure transparency, reproducibility, and adaptability. This design allows individual components, such as communication, task scheduling, and LLM-based code generation, to be independently extended or integrated into other robotic systems, facilitating community-driven development and long-term scalability.

Future work will scale trials to larger teams, extend application domains, and integrate continuous user feedback to further refine trust, usability, and long-term sustainability. Ultimately, FORMIGA advances collaborative autonomy by bridging high-level AI-driven planning with grounded human–robot interaction in the field.

## Data Availability

The original contributions presented in the study are included in the article/supplementary material, further inquiries can be directed to the corresponding author.

## Footnotes

1 FORMIGA, meaning “ant” in Portuguese, embodies the system’s qualities of strength, cooperation, and efficiency, mirroring the collaborative and resilient nature of ants in complex environments.

2
https://github.com/NVIDIA-ISAAC-ROS/isaac_ros_mission_client.

3
https://github.com/ROOSTER-fleet-management.

4
https://www.open-rmf.org/.

5
https://www.inorbit.ai/.

6
https://www.meilirobots.com/.

7
https://formant.io/.

8
https://groq.com/.

9
https://ferox.fbk.eu/.

10
https://github.com/Unity-Technologies/ROS-TCP-Connector.
